# Nutrition Composition and Single, 14-Day and 13-Week Repeated Oral Dose Toxicity Studies of the Leaves and Stems of *Rubus coreanus* Miquel

**DOI:** 10.3390/molecules21010065

**Published:** 2016-01-08

**Authors:** Ae-Son Om, Yu-Na Song, GeonMin Noh, HaengRan Kim, JeongSook Choe

**Affiliations:** 1 Laboratory of Food Safety and Toxicology, Department of Food Science and Nutrition, Hanyang University, Seoul 133-791, Korea; syn0878@gmail.com; 2 Functional Food and Nutrition Division, Department of Agrofood Resources, National Academy of Agricultural Science, Jeonbuk 565-851, Korea; team0708@korea.kr (G.M.N.); hrrkim@korea.kr (H.R.K.); swany@korea.kr (J.S.C.)

**Keywords:** *Rubus coreanus* Miquel leaves and stems, single dose toxicity, repeated dose toxicity, nutrition composition

## Abstract

The leaves and stems of the plant *Rubus coreanus* Miquel (RCMLS) are rich in vitamins, minerals and phytochemicals which have antioxidant, anti-hemolytic, anti-inflammatory, anti-fatigue and anti-cancer effects. However, RCMLS is not included in the Korean Food Standards Codex due to the lack of safety assurance concerning RCMLS. We evaluated single and repeated oral dose toxicity of RCMLS in Sprague-Dawley rats. RCMLS did not induce any significant toxicological changes in both male and female rats at a single doses of 2500 mg/kg/day. Repeated oral dose toxicity studies showed no adverse effects in clinical signs, body weight, food consumption, ophthalmic examination, urinalysis, hematology, serum biochemistry, necropsy findings, organ weight, and histopathology at doses of 625, 1250, and 2500 mg/kg/day. The LD_50_ and LOAEL of RCMLS might be over 2500 mg/kg body weight/day and no target organs were identified. Therefore, this study revealed that single and repeated oral doses of RCMLS are safe.

## 1. Introduction

*Rubus coreanus* Miquel (*R. coreanus*) is a deciduous broadleaf shrub of the *Rosaceae* family that originates from Korea and China. The unripe fruit of *R. coreanus*, commonly called *bokbunja* in Korea, contains various bioactive phenolic compounds such as flavonoids, anthocyanins, tannins, quercetin, as well as minerals, vitamins, *etc.* [1]. The unripe fruits of *R. coreanus* are used in traditional Korean herbal medicine to treat diabetes, spermatorrhea, enuresis, asthma and allergy-related illnesses [2,3]. With their high anthocyanin content the ripe fruits of *R. coreanus* are darker in color compared to other berries. They have an abundance of phenolic compounds such as ellagic acid, gallic acid, cinnamic acid, protocatechuic acid, sangiin H-4, sanguiin H-6, 23-hydroxytormentic acid, and nigaichgoside F [4,5,6,7]. They are reported to have antioxidant, antihemolytic, anticancer, anti-inflammatory, antibacterial, and anti-fatigue effects [8,9,10,11,12,13,14,15,16,17,18]. There is a general interest in identifying natural bioactive compounds, because these compounds have functional benefits such as regulating apoptosis in cancer and healthy cells [19].

Meanwhile, the leaves of *R. coreanus* are rich in tannins (ellagic acid, sanguiin H-5) and flavonoids (kaemferol, quercetin, quercetin 3-*O*-β-d-glucuronide-sodium salt, quercetin 3-*O*-β-d-glucuronide-sodium carboxylate) [20]. The stems of *R. coreanus* have epicatechin, catechin, procyanidin B-4 and sanguiin H-4 as well [21]. Moreover, the extract of *R. coreanus* leaves and stems (RCMLS) have been reported to have HMG-CoA reductase activity, nitric oxide generating, angiotensin converting enzyme, protein expression suppressing and endothelial NOS increasing effects, which are important factors in the cholesterol biosynthesis process [22,23]. *R. coreanus* has been consumed since the old times and its fruits, leaves and stems are known to possess physiological and pharmacological effects. RCMLS is also widely consumed in side dishes, teas, drinks or medical herbs in Korean folk medicine, but it is still not registered as a food material in the Korean Food Standards Codex. For food material registration, safety data obtained though toxicological testing is essential, but to date there are no safety studies on RCMLS. Therefore, this study aimed to provide a basis for the nutrition composition of RCMLS and to evaluate its single, 14 days and 13 weeks repeated oral dose toxicity in Sprague-Dawley rats.

## 2. Results and Discussion

### 2.1. Nutrition Compositon

The proximate composition, minerals and vitamins contents of RCMLS are shown in Table 1. Carbohydrates (76.81%) were the highest in content, which includes 66.42% fiber. Crude protein, fat, and moisture were 10.07%, 2.20%, and 5.57% on a dry weight basis, respectively.

**Table 1:** Nutrition compositions of the leaves and stems of *Rubus coreanus* Miquel.

Nutrient	Ingredient	Content	Ingredient	Content
Proximate composition (%)	Moisture	5.75	Carbohydrate ^1^	76.81
	Ash	5.17	Crude fat	2.20
	Crude protein	10.07		
Minerals (mg/100 g)	Ca	810.92	K	1463.96
	Mg	263.28	Cr	0.66
	Fe	13.57	Se	7.06 μg/100 g
	Zn	7.21	I	20.88 μg/100 g
	Cu	0.53	Mo	37.02 μg/100 g
	Mn	30.37		
Vitamins (mg/100 g)	B_1_	0.04	E	7.93
	B_2_	0.10	K	0.82
	B_6_	0.16	Niacin	0.51 mg NE/100 g
	D	0.07		

The contents were calculated by dry weight basis % (unit: %, weight/weight); ^1^ Carbohydrate includes 66.42% dietary fiber.

In mineral contents, K (1463.96 mg/100 g), Ca (810.92 mg/100 g), and Mg (263.28 mg/100 g) levels were high and Mn (30.37 mg/100 g), Fe (13.57 mg/100 g), Zn (7.21 mg/100 g) were detectable. In vitamins, vitamin E was the highest at 7.93 mg α-TE/100 g and the order of the remaining ones was vitamin K > niacin > vitamin B_6_ > vitamin B_2_ > vitamin D > vitamin B_1_.

Kim *et al.* [12] reported that fruits of *R. coreanus* fall in the range of 8%–10% moisture, 4%–5% crude protein, 3%–5% crude fat, 4%–7% ash, 12%–56% free sugar, and 11%–37% fiber on a dry weight basis. The mineral contents of *R. coreanus* were K (10,381–21,026 ppm), Ca (4976–11,489 ppm), Mg (2789–4446 ppm), P (2364–3425 ppm), Mg (151–200 ppm), Fe (32–37 ppm), Zn (15 ppm), Na (9–18 ppm) and Cu (7–8 ppm) on a dry weight basis. According to previous studies, the proximate composition of RCMLS is similar to that of fruits. Jung *et al.* [24] studied the proximate composition of each RCMLS component. The leaves showed 9.72% moisture, 1.75% crude protein and 1.75% crude fat and the stems showed 4.22%, 1.32% and 0.87%, respectively. However, research on the vitamin and fatty acid composition of RCMLS is nonexistent.

Table 2 lists the fatty acid composition of the RCMLS. α-linolenic acid was the highest component at 30.48%, followed by palmitic acid at 23.68% and linoleic acid at 15.51% on a dry weight basis. Total saturated fatty acid (SFA) was 39.32%, while monounsaturated fatty acid (MUFA) and polyunsaturated fatty acid (PUFA) were 4.21% and 46.72%, respectively. Thus, RCMLS contained more unsaturated fatty acid (UFA) than SFA. Meanwhile, Lee *et al.* [25] reported linoleic acid in the fatty acid composition of ripe fruits of *R. coreanus* was the highest at 3.573% and the ranking was linolenic acid > oleic acid > palmitic acid > arachidic acid > myristic acid > palmitoleic acid > stearic acid > caproic acid, and PUFA (70.78%) was higher than SFA (11.17%). The data in this study is thus not in accordance with the study conducted by Lee *et al.* [25] but UFA was higher than SFA regardless of whether fruits, leaves and stems were studied.

It is hard to compare the absolute content of the mixture of RCMLS as there are no study results analyzing the nutritional composition of the mixture. However, when compared to the previous studies [24] separately analyzing the leaves and stems, the mixture of RCMLS can be estimated to have similar ingredients.

**Table 2:** Fatty acid compositions of the leaves and stems of *Rubus coreanus* Miquel.

Fatty Acid	Content	Fatty Acid	Content
Lauric acid	1.64	Heneicosanoic acid	0.49
Myristic acid	1.01	Behenic acid	3.40
Palmitic acid	23.68	Arachidonic acid	0.73
Heptadecanoic acid	0.88	Lignoceric acid	3.41
Elaidic acid	0.53	ΣSFA	39.32
Oleic acid	4.21	ΣMUFA	4.21
Linoleic acid	15.51	ΣPUFA	46.72
Arachidic acid	4.81	ΣUFA	50.93
α-Linolenic acid	30.48		

The contents were calculated by dry weight basis (unit: %, weight/weight); Values are expressed as the area percentage of total fatty acid; Abbreviation; SFA: Saturated fatty acid, MUFA: Monounsaturated fatty acid, PUFA: Polyunsaturated fatty acid, UFA: Unsaturated fatty acid.

### 2.2. Single Oral Dose Toxicity Study

To investigate the toxicity and determine the lethal dose (LD_50_), RCMLS was administrated orally to male and female Sprague-Dawley (SD) rats at single doses of 0 (vehicle control) and 2500 mg/kg. During the 14 days experimental period, mortality, clinical signs, body weight and necropsy results were observed. No animal deaths or abnormal clinical signs were observed. In addition, there were no significant changes in body weight in the RCMLS-treated groups compared with the vehicle control group. No abnormalities were found during necropsy. Therefore, LD_50_ of RCMLS can be estimated to be over 2500 mg/kg body weight (b.w.).

### 2.3. 14 Days Repeated Oral Dose Toxicity Study

To determine the dosage of RCMLS for the 13 weeks repeated oral dose toxicity tests, male and female SD rats were orally fed with RCMLS for 14 days at doses of 0 (vehicle control), 625, 1250 and 2500 mg/kg/day. During the experimental period, mortality, clinical signs, body weight, food consumption, ophthalmic examination, urinalysis, hematology, serum biochemistry, necropsy, organ weight and histopathology were observed.

In the results, no animal deaths or abnormal clinical signs related to the administration of RCMLS were observed (Table 3). In addition, there were no significant body weight changes in RCMLS-treated groups compared with the vehicle control group (Table 4). Ophthalmic examination showed no abnormality in any test rats. Urinalysis and urine color observation showed no significant difference between male and female rats. Hematology tests showed no significant change caused by feeding RCMLS (Table 6).

Serum biochemistry test showed that K^+^ decreased in the males of the 2500 mg/kg/day group and Ca^2+^ and Na^+^ increased and Cl^−^ decreased in the females of the 2500 mg/kg/day group (Table 7). However, the range was very slight and within the biological change range [26]. Therefore, it was not considered toxicologically significant.

**Table 3:** Mortality and clinical sign of 14 days repeated oral dose and 13 weeks repeated oral dose toxicity studies of the leaves and stems of *Rubus coreanus* Miquel (%).

Group	S/N	Signs after 14 Days	S/N	Signs after 13 Weeks
Control	Male 0/5	None	Male 0/10	None
	Female 0/5	None	Female 0/10	None
625 mg/kg RCMLS	Male 0/5	None	Male 0/10	None
	Female 0/5	None	Female 1/10	Mass, right ventral neck
1250 mg/kg RCMLS	Male 0/5	None	Male 0/10	None
	Female 0/5	None	Female 0/10	None
2500 mg/kg RCMLS	Male 0/5	None	Male 0/10	None
	Female 0/5	None	Female 0/10	None

RCMLS: *Rubus coreanus* Miquel leaves and stems, S/N: Number of animals with the sign/Number of animals examined.

**Table 4:** Body weight of male and female rats in 14 days repeated oral dose toxicity study of the leaves and stems of *Rubus coreanus* Miquel.

Group	Sex	Body Weight (g)			Weight Gain (%)
		Day 0	Day 7	Day 14	
Control	Male	194.17 ± 5.37	258.98 ± 9.15	294.64 ± 17.73	52.6
	Female	163.66 ± 6.81	189.22 ± 14.95	217.95 ± 20.84	33.1
625 mg/kg RCMLS	Male	194.83 ± 8.09	261.06 ± 8.46	311.47 ± 11.27	60.9
	Female	161.21 ± 5.49	191.96 ± 10.10	214.23 ± 9.78	33.9
1250 mg/kg RCMLS	Male	195.53 ± 6.02	256.15 ± 10.90	296.68 ± 13.79	52.8
	Female	160.03 ± 7.09	190.03 ± 8.94	213.64 ± 7.89	33.5
2500 mg/kg RCMLS	Male	192.78 ± 6.59	243.43 ± 11.13	291.00 ± 18.51	51.0
	Female	163.08 ± 6.62	187.52 ± 9.01	207.01 ± 10.04	27.0

Data are presented as mean ± standard deviation; RCMLS: *Rubus coreanus* Miquel leaves and stems.

Absolute and relative organ weight showed no significant change in both male and females of all test groups (Table 9 and Table 10). Necropsy and histopathology test results showed miniaturized bilateral testes in the male of the 1250 mg/kg/day group that were confirmed as seminiferous tubules degeneration and the enlarged spleen in a female of the 2500 mg/kg/day group were confirmed to be increased cellularity of white pulp. However, it was not considered toxicologically significant because it was an isolated case and a natural occurrence.

In conclusion, when RCMLS was orally administered repeatedly for 14 days to SD rats, no systemic toxicological change in relation to RCMLS was observed. Therefore, setting a dosage as high as 2500 mg/kg/day in the subsequent 13 weeks repeated administration test seems appropriate.

### 2.4. 13 Weeks Repeated Oral Dose Toxicity Study

#### 2.4.1. Mortality, Clinical Signs, Body Weight and Food Consumption

There were no animal deaths during the experimental period, but a female of the 625 mg/kg/day group showed a subcutaneous mass in the right dorsal neck after 13 weeks of RCMLS administration until nucropsy (Table 3). However it was concluded as unrelated to RCMLS administration by necropsy and histopathology examination

Weight changes showed that on the 11–13 weeks, the weight of males in the 2500 mg/kg/day group decreased significantly compared to the vehicle control group (Table 5). These findings are concluded to be an effect of RCMLS but are within biological fluctuation range and other test items showed no support for adverse effect of RCMLS. Therefore, it was not considered toxicologically significant.

**Table 5:** Body weight of male and female rats in 13 weeks repeated oral dose toxicity study of the leaves and stems of *Rubus coreanus* Miquel.

Group	Sex	Body Weight (g)			Weight Gain (%)
		Week 0	Week 7	Week 13	
Control	Male	188.01 ± 7.26	477.50 ± 37.41	579.16 ± 56.03	208.0
	Female	142.95 ± 7.05	269.69 ± 22.30	313.66 ± 26.59	119.4
625 mg/kg RCMLS	Male	189.79 ± 6.70	495.16 ± 35.13	611.26 ± 43.19	222.1
	Female	142.39 ± 5.09	269.05 ± 26.70	311.62 ± 41.69	118.9
1250 mg/kg RCMLS	Male	190.20 ± 6.76	500.33 ± 45.79	614.66 ± 47.94	123.2
	Female	144.22 ± 7.73	274.73 ± 29.00	321.73 ± 39.22	123.1
2500 mg/kg RCMLS	Male	184.96 ± 12.72	445.67 ± 44.60	533.00 ± 54.44 **	188.2 **
	Female	141.71 ± 6.27	260.15 ± 22.28	296.31 ± 24.78	109.1

Data are presented as mean ± standard deviation; ** Significantly different from corresponding control value at *p* < 0.01; RCMLS: *Rubus coreanus* Miquel leaves and stems.

Food consumption measurement results showed 6–8 weeks period food consumption in the male of the 2500 mg/kg group and 12 weeks period in the female of the 2500 mg/kg group decreased significantly compared to the vehicle control group. However, these changes were transient and appeared to be unrelated to the doses or to the treatment with RCMLS*.*

#### 2.4.2. Ophthalmic Examination, Urinalysis, Hematology and Serum Biochemistry

No abnormalities were found in any groups in the ophthalmic examination and urinalysis.

Hematopoietic parameters are some of the most sensitive ones used to assess the toxicity of drugs in humans and animals, and a blood profile usually gives vital information on the response of the body to injury or stress [27,28]. In the present study, the hematology test results showed a significant decrease in mean platelet volume (MPV) in the males of the 2500 mg/kg/day group compared to the vehicle control group. Additionally, neutrophil (NE) and percent of neutrophil (NEP) in the females of the 625 mg/kg/day group significantly increased, whereas the percent of lymphocyte (LYP) decreased significantly. However, the difference was slight and within the biological fluctuation range [26], with no dose-dependency. Thus, it was concluded that RCMLS did not influence it.

Serum biochemistry result showed dose-dependency in total bilirubin and Mg^2+^ of the females of the 2500 mg/kg/day group and it significantly decreased when compared to the vehicle control group. Na^+^ in females of the 1250 and 2500 mg/kg/day group decreased significantly in a dose dependent manner (Table 8). But the changes were considered incidental, because they were very slight changes, not sex or dose-related, and unaccompanied by any correlative finding.

#### 2.4.3. Autopsy, Organ Weights and Histopathology

One case of cyst in the pituitary gland was observed in a male of the vehicle control group and one case of a mass in the right subcutaneous neck was observed in a female of the 625 mg/kg/day group. However, considering the histopathology test results, these findings were non dose-dependent and a natural occurrence or temporary and therefore, it was not considered toxicologically significant.

Absolute liver weights in the males of the 2500 mg/kg/day group, absolute right ovary weights in the females of the 2500 mg/kg/day group, relative both testes weights in males of the 625 and 1250 mg/kg/day group, and relative left adrenal gland weights in the males of the 1250 mg/kg/day group, decreased significantly when compared with those in the vehicle control group (Table 9 and Table 10).

**Table 6:** Hematological values of male and female rats in 14 days repeated oral dose and 13 weeks repeated oral dose toxicity studies of the leaves and stems of *Rubus coreanus* Miquel (%).

Group	Sex	HGB (g/dL)		HCT (%)		WBC (K/μL)		Platelet (K/μL)	
		14 Days	13 Weeks	14 Days	13 Weeks	14 Days	13 Weeks	14 Days	13 Weeks
Control	Male	14.7 ± 0.8	14.6 ± 0.9	45.5 ± 2.3	45.4 ± 2.5	7.01 ± 1.48	11.21 ± 3.49	1217 ± 90	990 ± 203
	Female	14.8 ± 1.0	15.1 ± 0.5	45.2 ± 2.5	45.9 ± 1.6	7.62 ± 2.66	6.83 ± 2.32	1310 ± 129	841 ± 98
625 mg/kg RCMLS	Male	14.1 ± 0.4	14.7 ± 0.6	44.3 ± 1.2	45.9 ± 1.9	8.22 ± 1.60	11.07 ± 2.85	1401 ± 117	1112 ± 73
	Female	15.4 ± 0.6	15.2 ± 0.5	46.5 ± 1.8	46.1 ± 1.2	10.77 ± 4.36	7.35 ± 1.29	1276 ± 197	947 ± 121
1250 mg/kg RCMLS	Male	14.3 ± 0.6	14.6 ± 0.4	44.7 ± 1.5	45.2 ± 1.4	8.15 ± 1.27	11.90 ± 2.00	1243 ± 113	1062 ± 143
	Female	15.2 ± 0.4	15.1 ± 0.5	45.7 ± 1.3	45.7 ± 1.5	8.63 ± 1.27	6.85 ± 1.78	1231 ± 102	925 ± 143
2500 mg/kg RCMLS	Male	14.0 ± 0.9	15.1 ± 0.3	43.6 ± 3.2	47.3 ± 0.8	8.55 ± 1.42	9.72 ± 1.90	1203 ± 146	1102 ± 151
	Female	14.8 ± 0.6	15.2 ± 0.6	44.8 ± 1.4	45.9 ± 1.6	7.88 ± 0.93	6.87 ± 1.96	1314 ± 295	965 ± 115
**Group**	**Sex**	**MP** **V** **(fL)**		**NE (** **K** **/** **μ** **L)**		**NEP (** **%** **)**		**L** **Y** **P (** **%** **)**	
		**14 Days**	**13 Weeks**	**14 Days**	**13 Weeks**	**14 Days**	**13 Weeks**	**14 Days**	**13 Weeks**
Control	Male	5.5 ± 0.8	4.7 ± 0.3	1.04 ± 0.30	2.16 ± 1.17	15.6 ± 6.6	19.0 ± 7.5	79.6 ± 7.3	76.1 ± 8.5
	Female	5.6 ± 0.5	4.6 ± 0.2	0.75 ± 0.18	0.99 ± 0.34	10.7 ± 3.7	15.0 ± 5.0	85.4 ± 3.9	79.9 ± 5.0
625 mg/kg RCMLS	Male	5.7 ± 1.2	4.6 ± 0.1	1.23 ± 0.24	2.00 ± 1.23	15.5 ± 4.1	17.6 ± 9.1	80.6 ± 3.9	77.8 ± 9.9
	Female	4.8 ± 0.5	4.5 ± 0.3	0.84 ± 0.48	1.55 ± 0.58 **	8.5 ± 4.3	20.7 ± 5.6 **	87.2 ± 3.8	74.1 ± 6.0 **
1250 mg/kg RCMLS	Male	5.4 ± 0.8	4.7 ± 0.2	1.08 ± 0.25	2.06 ± 0.60	13.3 ± 2.9	17.4 ± 4.5	82.6 ± 2.6	77.8 ± 4.6
	Female	5.2 ± 0.5	4.6 ± 0.2	0.86 ± 0.17	0.79 ± 0.30	10.1 ± 2.3	12.3 ± 5.3	86.2 ± 3.0	82.3 ± 5.8
2500 mg/kg RCMLS	Male	5.8 ± 1.0	4.5* ± 0.1	1.24 ± 0.67	1.81 ± 0.68	14.4 ± 6.7	19.0 ± 7.5	81.6 ± 7.8	76.4 ± 7.6
	Female	4.9 ± 0.7	4.5 ± 0.2	0.82 ± 0.16	0.95 ± 0.39	10.4 ± 1.5	14.2 ± 4.6	85.1 ± 1.3	81.7 ± 4.7

Data are presented as mean ± standard deviation; ** Significantly different from corresponding control value at *p* < 0.01; RCMLS: *Rubus coreanus* Miquel leaves and stems, HCT: hematocrit, HGB: hemoglobin, WBC: white blood corpuscles, MPV: mean platelet volume, NE: neutrophil, NEP; percent of neutrophil, LYP: percent of lymphocyte.

**Table 7:** Serum biochemistry in 14 days repeated oral dose toxicity study of the leaves and stems of *Rubus coreanus* Miquel.

Parameter	Control	625 mg/kg RCMLS	1250 mg/kg RCMLS	2500 mg/kg RCMLS
Male				
Glucose (mg/dL)	128 ± 29	128 ± 17	145 ± 18	158 ± 8
BUN (mg/dL)	13.1 ± 1.6	12.5 ± 1.1	14.2 ± 1.6	12.1 ± 2.5
Creatinine (mg/dL)	0.38 ± 0.04	0.32 ± 0.05	0.39 ± 0.04	0.35 ± 0.03
Total protein (g/dL)	6.0 ± 0.4	6.0 ± 0.2	5.9 ± 0.2	5.7 ± 0.2
Albumin (g/dL)	2.4 ± 0.2	2.3 ± 0.2	2.3 ± 0.1	2.3 ± 0.2
A/G ratio	0.66 ± 0.03	0.62 ± 0.05	0.65 ± 0.04	0.65 ± 0.05
Total bilirubin (mg/dL)	0.01 ± 0.01	0.02 ± 0.01	0.01 ± 0.01	0.00 ± 0.01
LDH (IU/L)	1706 ± 631	1777 ± 923	1754 ± 689	1793 ± 761
Cholesterol (mg/dL)	62 ± 7	68 ± 16	73 ± 14	68 ± 16
Triglycerides (mg/dL)	28 ± 7	42 ± 14	34 ± 14	35 ± 15
AST (U/L)	158 ± 28	166 ± 48	167 ± 43	165 ± 36
ALT (U/L)	43 ± 4	39 ± 8	44 ± 6	44 ± 6
ALP (U/L)	669 ± 114	598 ± 126	654 ± 108	761 ± 59
Mg(mg/dL)	2.9 ± 0.3	2.8 ± 0.1	2.9 ± 0.1	2.7 ± 0.1
Ca(mg/dL)	10.0 ± 0.2	9.9 ± 0.1	9.7 ± 0.4	9.7 ± 0.3
Na(mmol/L)	139 ± 3	138 ± 2	139 ± 4	137 ± 3
K(mmol/L)	6.1 ± 0.4	5.9 ± 0.2	5.8 ± 0.3	5.4 ± 0.4 *
Female				
Glucose (mg/dL)	184 ± 34	198 ± 36	193 ± 23	197 ± 26
BUN (mg/dL)	14.8 ± 2.0	14.2 ± 2.4	13.9 ± 2.8	14.7 ± 1.7
Creatinine (mg/dL)	0.58 ± 0.11	0.60 ± 0.13	0.59 ± 0.11	0.53 ± 0.13
Total protein (g/dL)	6.7 ± 0.5	6.6 ± 0.3	7.0 ± 0.3	6.7 ± 0.7
Albumin (g/dL)	2.8 ± 0.3	2.7 ± 0.1	2.9 ± 0.2	2.7 ± 0.4
A/G ratio	0.71 ± 0.05	0.69 ± 0.03	0.71 ± 0.08	0.69 ± 0.07
Total bilirubin (mg/dL)	0.09 ± 0.05	0.08 ± 0.03	0.06 ± 0.02	0.03 ± 0.01
LDH (IU/L)	600 ± 476	579 ± 353	420 ± 327	481 ± 426
Cholesterol (mg/dL)	85 ± 18	72 ± 16	91 ± 23	84 ± 20
Triglycerides (mg/dL)	23 ± 11	19 ± 7	31 ± 15	22 ± 11
AST (U/L)	113 ± 43	103 ± 33	87 ± 16	90 ± 15
ALT (U/L)	42 ± 23	36 ± 22	32 ± 8	29 ± 5
ALP (U/L)	140 ± 34	163 ± 38	130 ± 37	174 ± 50
Mg(mg/dL)	2.9 ± 0.1	2.8 ± 0.1	2.7 ± 0.2	2.8 ± 0.2
Ca(mg/dL)	9.9 ± 0.2	9.8 ± 0.2	10.0 ± 0.4	10.5 ± 0.5 *
Na(mmol/L)	134 ± 2	135 ± 5	136 ± 3	142 ± 1 **
K(mmol/L)	5.8 ± 0.7	6.1 ± 0.2	6.2 ± 0.2	6.0 ± 0.3

Data are presented as mean ± standard deviation; * Significantly different from corresponding control value at *p* < 0.05; ** Significantly different from corresponding control value at *p* < 0.01; RCMLS: *Rubus coreanus* Miquel leaves and stems, ALP: alkaline phosphatase, ALT: alanine transaminase; AST: aspartate transaminase, A/G ratio: Albumin/Globulin ratio, LDH: lactate dehydrogenase, BUN: blood urea nitrogenm Mg: magnesium, Ca: calcium, Na: sodium, K: potassium.

**Table 8:** Serum biochemistry in 13 weeks repeated oral dose toxicity study of the leaves and stems of *Rubus coreanus* Miquel.

Parameter	Control	625 mg/kg RCMLS	1250 mg/kg RCMLS	2500 mg/kg RCMLS
Male				
Glucose (mg/dL)	184 ± 34	198 ± 36	193 ± 23	197 ± 26
BUN (mg/dL)	14.8 ± 2.0	14.2 ± 2.4	13.9 ± 2.8	14.7 ± 1.7
Creatinine (mg/dL)	0.58 ± 0.11	0.60 ± 0.13	0.59 ± 0.11	0.53 ± 0.13
Total protein (g/dL)	6.7 ± 0.5	6.6 ± 0.3	7.0 ± 0.3	6.7 ± 0.7
Albumin (g/dL)	2.8 ± 0.3	2.7 ± 0.1	2.9 ± 0.2	2.7 ± 0.4
A/G ratio	0.71 ± 0.05	0.69 ± 0.03	0.71 ± 0.08	0.69 ± 0.07
Total bilirubin (mg/dL)	0.09 ± 0.05	0.08 ± 0.03	0.06 ± 0.02	0.03 ± 0.01
LDH (IU/L)	600 ± 476	579 ± 353	420 ± 327	481 ± 426
Cholesterol (mg/dL)	85 ± 18	72 ± 16	91 ± 23	84 ± 20
Triglycerides (mg/dL)	23 ± 11	19 ± 7	31 ± 15	22 ± 11
AST (U/L)	113 ± 43	103 ± 33	87 ± 16	90 ± 15
ALT (U/L)	42 ± 23	36 ± 22	32 ± 8	29 ± 5
ALP (U/L)	140 ± 34	163 ± 38	130 ± 37	174 ± 50
Mg(mg/dL)	2.6 ± 0.2	2.6 ± 0.2	2.5 ± 0.2	2.6 ± 0.2
Ca(mg/dL)	10.0 ± 0.3	10.0 ± 0.2	10.0 ± 0.2	10.0 ± 0.3
Na(mmol/L)	141 ± 5	143 ± 5	142 ± 4	142 ± 4
K(mmol/L)	5.4 ± 0.7	5.4 ± 0.4	5.5 ± 0.4	5.8 ± 0.9
Female				
Glucose (mg/dL)	192 ± 32	212 ± 17	210 ± 19	206 ± 22
BUN (mg/dL)	13.8 ± 1.7	14.5 ± 1.7	13.3 ± 1.8	14.7 ± 1.8
Creatinine (mg/dL)	0.44 ± 0.04	0.48 ± 0.03	0.46 ± 0.03	0.46 ± 0.05
Total protein (g/dL)	6.3 ± 0.3	6.3 ± 0.2	6.3 ± 0.3	6.2 ± 0.4
Albumin (g/dL)	2.3 ± 0.1	2.3 ± 0.1	2.3 ± 0.1	2.3 ± 0.1
A/G ratio	0.56 ± 0.04	0.57 ± 0.03	0.58 ± 0.05	0.59 ± 0.03
Total bilirubin (mg/dL)	0.03 ± 0.05	0.04 ± 0.02	0.02 ± 0.02	0.03 ± 0.02
LDH (IU/L)	649 ± 619	944 ± 526	763 ± 544	646 ± 676
Cholesterol (mg/dL)	69 ± 24	67 ± 15	73 ± 15	65 ± 11
Triglycerides (mg/dL)	35 ± 15	39 ± 21	47 ± 19	32 ± 15
AST (U/L)	109 ± 28	119 ± 29	109 ± 30	105 ± 26
ALT (U/L)	39 ± 9	39 ± 7	35 ± 6	37 ± 7
ALP (U/L)	225 ± 35	225 ± 44	195 ± 39	234 ± 44
Mg(mg/dL)	2.7 ± 0.1	2.7 ± 0.2	2.5 ± 0.2	2.5 ± 0.1 *
Ca(mg/dL)	10.2 ± 0.8	10.1 ± 0.4	10.5 ± 0.6	10.2 ± 0.5
Na(mmol/L)	140 ± 2	138 ± 3	136 ** ± 3	135 ± 4 **
K(mmol/L)	5.7 ± 0.9	5.4 ± 0.5	5.6 ± 0.7	5.9 ± 0.4

Data are presented as mean ± standard deviation; * Significantly different from corresponding control value at *p* < 0.05; ** Significantly different from corresponding control value at *p* < 0.01; RCMLS: *Rubus coreanus* Miquel leaves and stems, ALP: alkaline phosphatase, ALT: alanine transaminase; AST: aspartate transaminase, A/G ratio: Albumin/Globulin ratio, LDH: lactate dehydrogenase, BUN: blood urea nitrogenm Mg: magnesium, Ca: calcium, Na: sodium, K: potassium.

**Table 9:** Absolute organ weights of male and female rats in 14 days repeated oral dose and 13 weeks repeated oral dose toxicity studies of the leaves and stems of *Rubus coreanus* Miquel.

Group	Sex	Organ Weight (mg/kg/Day)											
		Liver		Adrenal (Left)		Adrenal (Right)		Testis/Ovary (Left)		Testis/Ovary (Right)		Spleen	
		14 Days	13 Weeks	14 Days	13 Weeks	14 Days	13 Weeks	14 Days	13 Weeks	14 Days	13 Weeks	14 Days	13 Weeks
Control	Male	8.8070 ± 0.6145	14.4278 ± 2.2296	0.0275 ± 0.0033	0.0329 ± 0.0065	0.0247 ± 0.0018	0.0307 ± 0.0082	1.4477 ± 0.1851	1.9374 ± 0.1253	1.4947 ± 0.1887	1.9352 ± 0.1137	0.7522 ± 0.1046	1.0541 ± 0.1852
	Female	6.4639 ± 1.1599	7.6186 ± 0.9044	0.0336 ± 0.0042	0.0353 ± 0.0054	0.0275 ± 0.0049	0.0362 ± 0.0055	0.0504 ± 0.0151	0.0445 ± 0.0089	0.0431 ± 0.0120	0.0480 ± 0.0088	0.4818 ± 0.0517	0.5485 ± 0.0661
625 mg/kg RCMLS	Male	9.5767 ± 0.9401	14.5220 ± 1.4821	0.0263 ± 0.0048	0.0300 ± 0.0055	0.0242 ± 0.0040	0.0275 ± 0.0046	1.4641 ± 0.0960	1.8488 ± 0.1465	1.4779 ± 0.0567	1.8611 ± 0.1518	0.6861 ± 0.0548	1.0005 ± 0.1286
	Female	6.1001 ± 0.2304	7.3090 ± 1.0428	0.0322 ± 0.0049	0.0363 ± 0.0086	0.0305 ± 0.0049	0.0331 ± 0.0057	0.0418 ± 0.0052	0.0470 ± 0.0099	0.0389 ± 0.0065	0.0475 ± 0.0068	0.4437 ± 0.0598	0.6002 ± 0.0768
1250 mg/kg RCMLS	Male	8.3576 ± 0.4890	15.239 ± 1.7837	0.0221 ± 0.0044	0.0270 ± 0.0074	0.0205 ± 0.0049	0.0284 ± 0.0042	1.2772 ± 0.3645	1.8611 ± 0.1288	1.2739 ± 0.3564	1.8696 ± 0.1363	0.6647 ± 0.1423	0.9661 ± 0.1545
	Female	6.4504 ± 0.8888	7.6644 ± 0.7581	0.0343 ± 0.0054	0.0329 ± 0.0067	0.0332 ± 0.0054	0.0329 ± 0.0051	0.0456 ± 0.0119	0.0487 ± 0.0087	0.0395 ± 0.0026	0.0452 ± 0.0060	0.4856 ± 0.0703	0.5770 ± 0.1031
2500 mg/kg RCMLS	Male	8.5526 ± 0.7034	12.5166 ± 1.6351 *	0.0250 ± 0.0073	0.0296 ± 0.0042	0.0253 ± 0.0065	0.0291 ± 0.0044	1.3956 ± 0.1045	1.8965 ± 0.0922	1.3873 ± 0.1331	1.9431 ± 0.0692	0.7834 ± 0.2388	0.8626 ± 0.1287
	Female	6.2359 ± 0.5127	6.9086 ± 0.8667	0.0330 ± 0.0028	0.0316 ± 0.0032	0.0311 ± 0.0035	0.0308 ± 0.0038	0.0409 ± 0.0041	0.0368 ± 0.0101	0.0411 ± 0.0092	0.0395 * ± 0.0061	0.5028 ± 0.0881	0.5510 ± 0.0585

Data are presented as mean ± standard deviation; * Significantly different from corresponding control value at *p* < 0.05; RCMLS: *Rubus coreanus* Miquel leaves and stems.

**Table 10:** Relative organ weights of male and female rats in 14 days repeated oral dose and 13 weeks repeated oral dose toxicity studies of the leaves and stems of *Rubus coreanus* Miquel.

Group	Sex	Organ Weight (mg/kg/Day)											
		Liver		Adrenal (Left)		Adrenal (Right)		Testis/Ovary (Left)		Testis/Ovary (Right)		Spleen	
		14 Days	13 Weeks	14 Days	13 Weeks	14 Days	13 Weeks	14 Days	13 Weeks	14 Days	13 Weeks	14 Days	13 Weeks
Control	Male	3.2112 ± 0.0775	2.6216 ± 0.2166	0.0100 ± 0.0008	0.0061 ± 0.0015	0.0090 ± 0.0005	0.0057 ± 0.0016	0.5283 ± 0.0659	0.3565 ± 0.0414	0.5452 ± 0.0632	0.3559 ± 0.0381	0.2743 ± 0.0348	0.1919 ± 0.0287
	Female	3.2578 ± 0.3255	2.6021 ± 0.2819	0.0170 ± 0.0015	0.0121 ± 0.0019	0.0141 ± 0.0032	0.0124 ± 0.0023	0.0253 ± 0.0054	0.0152 ±0.0032	0.0218 ± 0.0052	0.0166 ± 0.0042	0.2445 ± 0.0208	0.1876 ± 0.0225
625 mg/kg RCMLS	Male	3.3865 ± 0.2391	2.4920 ± 0.1050	0.0093 ± 0.0013	0.0052 ± 0.0011	0.0086 ± 0.0012	0.0047 ± 0.0008	0.5188 ± 0.0362	0.3190 ± 0.0329 **	0.5239 ± 0.0278	0.3212 ± 0.0342 **	0.2427 ± 0.0122	0.1722 ± 0.0217
	Female	3.1429 ± 0.1913	2.5051 ± 0.1380	0.0166 ± 0.0031	0.0125 ± 0.0027	0.0158 ± 0.0030	0.0115 ± 0.0022	0.0215 ± 0.0028	0.0164 ± 0.0041	0.0200 ± 0.0034	0.0165 ± 0.0031	0.2280 ± 0.0276	0.2079 ± 0.0315
1250 mg/kg RCMLS	Male	3.1119 ± 0.0836	2.5951 ± 0.1412	0.0082 ± 0.0015	0.0046 ± 0.0011 *	0.0076 ± 0.0016	0.0049 ± 0.0006	0.4734 ± 0.1241	0.3194 ± 0.0333 **	0.4720 ± 0.1201	0.3209 ± 0.0345 **	0.2471 ± 0.0493	0.1649 ± 0.0228
	Female	3.3197 ± 0.3297	2.5263 ± 0.1478	0.0177 ± 0.0023	0.0108 ± 0.0019	0.0171 ± 0.0021	0.0108 ± 0.0014	0.0234 ± 0.0055	0.0164 ± 0.0043	0.0204 ± 0.0010	0.0152 ± 0.0036	0.2508 ± 0.0358	0.1892 ± 0.0221
2500 mg/kg RCMLS	Male	3.2466 ± 0.1202	2.4603 ± 0.1485	0.0094 ± 0.0024	0.0059 ± 0.0010	0.0095 ± 0.0021	0.0058 ± 0.0009	0.5298 ± 0.0123	0.3765 ± 0.0394	0.5264 ± 0.0272	0.3856 ± 0.0379	0.2955 ± 0.0797	0.1697 ± 0.0193
	Female	3.3406 ± 0.1756	2.4934 ± 0.1470	0.0177 ± 0.0015	0.0115 ± 0.0009	0.0167 ± 0.0023	0.0111 ± 0.0012	0.0219 ± 0.0019	0.0134 ± 0.0040	0.0221 ± 0.0051	0.0144 ± 0.0025	0.2702 ± 0.0497	0.2001 ± 0.0216

Data are presented as mean ± standard deviation; * Significantly different from corresponding control value at *p* < 0.05; ** Significantly different from corresponding control value at *p* < 0.01; RCMLS: *Rubus coreanus* Miquel leaves and stems.

However, it was slight and within the biological fluctuation range and did not show dose-dependency. Additionally, no clear relative change in absolute and relative organ weight was found. Therefore, it was not considered toxicologically significant. At the histopathology test, in a male of the vehicle control group, the cyst observed in the pituitary gland was confirmed as a nerve cyst. Also, the mass observed in the subcutaneous dorsal neck in a female of the 625 mg/kg/day group was confirmed as an adenocarcinoma in the mammary gland. However, no metastasis findings were observed, there was no dose-dependency, and they are sometimes naturally observed in the female mammary gland.

The histopathology test of the lungs showed inflammation of the terminal bronchiole and alveolar duct and foreign body granuloma. Inflammation was found to be somewhat dose-dependent in both the male and female test substance administered groups. However, the vehicle control group also showed inflammation. Considering that it was found near terminal bronchiole and alveolar duct, and the foreign body granuloma covered in sporadic brown foreign substance was found in some individuals, and administered dosage was at 20 mL/kg, which was quite high and formed much froth on preparation, the findings were evaluated to have caused by aspiration during administration. Therefore, it does not seem to be a side effect of RCMLS consumption. Including the osseous metaplasia of the lungs found in a male of the vehicle control group, the rest of the female and male of the vehicle control group, and 2500 mg/kg/day group findings were either observed in the control group, were sporadic, or were natural occurrences. Therefore, it was not considered toxicologically significant.

In conclusion, as the result of repeated 13 weeks oral administration of RCMLS, no systemic toxicological changes were observed. Therefore, the no observed adverse effect level (NOAEL) of RCMLS was set at 2500 mg/kg/day and no target organs were observed.

## 3. Experimental Section

### 3.1. Preparation of the Leaves and Stems of Rubus coreanus Miquel

RCMLS was collected in May 2014 from the Gochang Bokbunja Research Institute, Gochang, South Korea. The dried leaves and stems were mixed in the same percentage and pulverized using a Pin-type Mill (Seichin, Tokyo, Japan). The pulverized sample was stored at 4 °C prior to use. RCMLS was prepared by suspending the powder with sterile water for injection according to the doses assigned to each group in the single, 14 days and 13 weeks repeated oral dose toxicity studies.

### 3.2. Nutrition Composition

Proximate composition analyses of RCMLS were done for moisture, ash, crude protein, crude fat, carbohydrate and fiber contents according to the methods described by the Korean Food Standards Codex [29] and Korean Health Functional Food Code [30]. Moisture was determined by oven-drying at 105 °C to constant weight. Ash content was determined gravimetrically in a muffle furnace by heating at 550 °C to constant weight. Crude fat content was determined using the decomposition method. Crude protein (6 × 6.25) was determined on a LECO^®^ TruSpec^®^ CN (Carbon/Nitrogen Determinator, St. Joseph, MI, USA). Carbohydrate content was calculated as the difference between 100 and the sum of the moisture, crude protein, crude fat and ash contents. The dietary fiber content was obtained as an indigestible residue after enzymatic digestion of non-dietary fiber components.

Mineral, vitamin contents and fatty acid composition of RCMLS were done according to the methods described by the Korean Health Functional Food Code using inductive coupled plasma (ICP, Teledyne Leeman Labs, Hudson, NH, USA), Nanospace SI-2 HPLC system (Shiseido Co., Ltd., Tokyo, Japan) and Agilent 7890 GC system (Agilent Technologies, Santa Clara, CA, USA), respectively.

### 3.3. Experimental Animals

Male and female specific pathogene free SD rats (160–220 g) were obtained from the animal facility of Orient Bio (Sungnam, Korea). In case of the females, only ones that were not pregnant or had ever given birth were selected. The rats used in this study are widely used in micronuclear tests, as it is a suitable test animal in toxicology experiments. Also this strain of rat has much accumulated base test data, which can be used in test result interpretation and evaluation. The animals were starved, with exception to water, from the night before the forced oral administration using a probe to empty stomach contents. After 3–4 h food was redistributed. The animals were housed in a room maintained at a temperature of 23.2 ± 0.3 °C and 23.2 ± 0.8 °C, and a relative humidity of 51.4% ± 3.4% and 51.4% ± 5.4% for the 14 days single dose and repeated dose toxicity, respectively. The animals were housed in a room maintained at a temperature of 22.9 ± 0.7 °C and a relative humidity of 47.9% ± 3.9% during the 13 weeks repeated dose toxicity study. They had a 12 h dark/light cycle. Procedures involving animals and their care were approved by the ethics committee, Korea Conformity Laboratories, 2014.

### 3.4. Study Design Overview

This study was conducted in compliance with the Good Laboratory Practice (GLP) and Test Guidelines of the Organization for Economic Cooperation and Development (OECD) [31,32], and the Korea Food and Drug Administration (KDFA) at the GLP institute approved by the KFDA [33,34].

#### 3.4.1. Single Oral dose Toxicity Study

SD rats of both male and female were divided into two groups (I, II) of 22 rats (11 males and 11 females) matched for weight. All animals had free access to water and food throughout the experimental period. Group II was administrated RCMLS at single oral doses of the 2500 mg/kg, and Group I served as the vehicle control, receiving the same volume of sterile water. Animals were starved, with exception to water, from the night before the forced oral administration using a probe to empty stomach contents, and food was offered approximately 4 h after administration. Dosing volume was set to 20 mL/kg for toxicity study, based on the most recent body weight.

Clinical signs of animals were continuously monitored during the first 24 h and then daily thereafter, for a total period of 14 days. Changes in the normal activity of rats were monitored and the time at which signs of toxicity or death appeared was documented. On administration day, test animals were observed after 30 min of administration and every 6 h thereafter. Clinical signs observation was conducted until 14 days after administration. Changes in the body weights of the rats were monitored. At the end of the observation period, all surviving animals were fasted overnight.

After 14 days of administration, all surviving rats were anesthetized with CO_2_ gas to open their abdomen. Then they were killed by severing the caudal vena cava and the abdominal artery for bloodletting to observe the organs with the naked eye.

#### 3.4.2. 14 Days Repeated Oral dose Toxicity Study

Four groups of 22 animals (11 males and 11 females) were used. Groups II-IV received RCMLS at daily doses of 625 mg/kg/day, 1250 mg/kg/day and 2500 mg/kg/day, and Group I served as the vehicle control receiving the same volume of distilled water, for a period of 14 days. Animals underwent forced oral administration using a probe to empty stomach contents. Dosing volume was set to 20 mL/kg/day for the toxicity study, based on the most recent body weight. During the period of administration, the animals were weighed and observed daily to detect any clinical signs of toxicity. After 14 days, all surviving animals were investigated in the same way used in the single oral dose toxicity study.

Additionally, food consumption measurements were taken at initial administration and once weekly. Method of measurement was, food distribution amount on the day before weigh-in was recorded and then the remaining food amount on the day of weigh-in was used to calculate 1 day food consumption amount. Average consumption volume (g/rat/day) was calculated per individual animal. Appearance of animals’ eyes was observed at group division and on the last week of administration precedents of the vehicle control group and high dosage group (Group IV, 2500 mg/kg/day) were observed by the naked eye.

During the last administration week, five rats from each test group were transferred to a metabolic cage, where fresh urine was collected and tested for following items using urine screening test (Siemens) and urine auto-analyzer (CliniTek 50, Siemens/Bayer, Munich, Germany; MAI-050-01). Urine tone was observed with the naked eye.

Hematology test was conducted using a blood analyzer (ADVIA 2120, Siemens; MAI-105-01). Planned autopsy rats were starved overnight, anesthetized with CO_2_ gas, then blood was collected from ventral aorta through laparoscopy. EDTA-2K anticoagulant was used.

For serum biochemistry test, blood chemical testing device (Hitachi 7180, Hitachi, Tokyo, Japan; MAI-059-01) was used. The serum used derived from the blood collected from abdominal aorta at planned autopsy was centrifuged for 10 min at 3000 rpm.

After the last administration and autopsy, the following organs were extracted and weighed on an electronic scale. All following organs listed below were fixed with 10% sterile formalin solution: thymus, spleen, pancreas, stomach, duodenum, jejunum, ileum, cecum, colon, rectum, mesenteric lymph nodes, mandibular lymph nodes, salivary gland, thyroid gland (including parathyroid gland), Harderian gland, heart, lung, kidneys, adrenal glands, liver, aorta, brain, pituitary gland, tongue, trachea, esophagus, sternum, thoracic spinal cord, femorotibial joint, peripheral nerve (sciatic), skeletal muscle (femoral), prostate gland, seminal vesicles, ovaries, uterus, vagina, urinary bladder, and skin (including mammary gland). The testes were fixed in Bouin’s solution and the eyes in Davidson’s solution.

#### 3.4.3. 13 Weeks Repeated Oral dose Toxicity Study

Four groups of 88 rats (44 males and 44 females) were used. Groups II-IV received RCMLS at daily doses of 625, 1250 and 2500 mg/kg/day, and Group I served as the vehicle control receiving distilled water at the same volume, for a period of 13 weeks. During the period of administration, the animals were weighed and observed daily to detect any signs of toxicity. After 13 weeks, all surviving animals were investigated in the same way that was used in the subchronic toxicity study of 14 days repeated oral dose.

### 3.5. Statistical Analysis

Data are expressed as the means ± standard deviation and analyzed by a one-way analysis of variance (ANOVA). When significant differences existed, Duncan’s multiple range test was used to compare the means. SPSS 12.0 K (SPSS Inc, Chicago, IL, USA) was used for all statistical analysis. A *p<* 0.05 or 0.01 were considered as statistically different.

## 4. Conclusions

To investigate the possible application of RCMLS as food materials, we carried out an analysis of the nutritional composition and toxicity of RCMLS following 14 days single and repeated oral administration and 13 weeks of repeated oral administration to SD rats. The proximate composition of RCMLS was 76.81% carbohydrate, 10.07% crude protein, 5.57% moisture, 5.17% crude ash and 2.20% crude fat, respectively. RCMLS contained high contents of minerals and vitamins such as K and E, respectively. The major fatty acid components were 30.48% α-linolenic acid, 23.68% palmitic acid and 15.51% linoleic acid. Single and repeated oral administration of RCMLS produced no significant toxic effects in SD rats. Under the present experimental conditions, the approximate LD_50_ of RCMLS might be over 2500 mg/kg b.w for both male and female, the NOAEL might be over 2500 mg/kg/day for both male and female, and no target organs were identified.
